# Tractography of the prostatic neurovascular bundles: technique and interpretation

**DOI:** 10.1186/1470-7330-15-S1-P33

**Published:** 2015-10-02

**Authors:** A Lakhani, T Barwick, W Gedroyc, I Lavdas, N Ngo, A Rockall, J Vale, M Winkler

**Affiliations:** 1 Imperial College London, Exhibition Road, London, SW7 2AZ, UK

## Learning objectives

To be familiar with the diffusion tensor sequence acquisition used to delineate the tracts of the prostatic neurovascular bundles.

To understand the method for seeding and identifying the tracts and displaying the anatomy for surgical planning.

To be familiar with the potential imaging and interpretation pitfalls.

## Content organisation

Diffusion tensor imaging (DTI) may be used for tractography of the peri-prostatic neurovascular bundles (NVBs). We will describe the MR acquisition technique that may be used to demonstrate the NVB’s. The method used for seeding the tracts will be illustrated with examples in healthy volunteers and in cases of prostate cancer. Correlation of the tractography with the histological sites of NVB’s will be demonstrated. Pitfalls related to the MR acquisition will be discussed and potential difficulties with interpretation will be presented.

## Conclusion

Tractography of the prostatic NVB’s is feasible and has the potential to demonstrate the anatomical position of the NVBs in relation to prostate cancer. This information could in future influence surgical decision-making.

